# Expression of Olig2, Nestin, NogoA and AQP4 have no impact on overall survival in IDH-wildtype glioblastoma

**DOI:** 10.1371/journal.pone.0229274

**Published:** 2020-03-11

**Authors:** Felix Behling, Alonso Barrantes-Freer, Carl Ludwig Behnes, Florian Stockhammer, Veit Rohde, Antonia Adel-Horowski, Odir Antonio Rodríguez-Villagra, Miguel Angel Barboza, Wolfgang Brück, Ulrich Lehmann, Christine Stadelmann, Christian Hartmann

**Affiliations:** 1 Institute of Neuropathology, University Medical Center Goettingen, Goettingen, Germany; 2 Department of Neurosurgery, University Hospital Tuebingen, Tuebingen, Germany; 3 Center for CNS Tumors, Comprehensive Cancer Center Tuebingen-Stuttgart, University Hospital Tuebingen, Tuebingen, Germany; 4 Department of Neuropathology, Leipzig University Medicine, Leipzig, Germany; 5 Institute of Pathology, University Medical Center Goettingen, Goettingen, Germany; 6 Department of Neurosurgery, University Medical Center Goettingen, Goettingen, Germany; 7 Neuroscience Research Center, University of Costa Rica, San José, Costa Rica; 8 Institute for Psychological Research, University of Costa Rica, San José, Costa Rica; 9 Neurosciences Department, Hospital Dr. Rafael A. Calderón Guardia, CCSS, University of Costa Rica, San José, Costa Rica; 10 Institute of Pathology, Hannover Medical School, Hannover, Germany; 11 Department of Neuropathology, Institute of Pathology, Hannover Medical School, Hannover, Germany; University of Alabama at Birmingham, UNITED STATES

## Abstract

Despite many years of research efforts and clinical trials the prognosis of patients diagnosed with glioblastoma remains very poor. The oligodendrocyte transcription factor 2 (Olig2) was identified as a marker for glioma stem cells, which are believed to be responsible for glioma recurrence and therapy resistance. In this retrospective analysis we assessed the prognostic value of oligodendroglial and glioma stem cell markers in 113 IDH-wildtype glioblastomas. Immunohistochemical staining for Olig2, NogoA, AQP4 and Nestin was performed in combination with sequencing of *IDH1* and *IDH2* as well as promotor methylation analysis of the *MGMT* gene. Even though differences in overall survival according to Olig2 expression were observed, univariate and multivariate survival analysis did not reveal a firm significant prognostic impact of Olig2, NogoA, AQP4 or Nestin expression. Additionally, no differences in the expression of these markers depending on clinical status, age or gender were found. The established independent prognostic factors age<65, Karnofsky Performance Status> = 70 and methylated *MGMT* gene promoter were significant in the multivariate analysis. In conclusion expression of oligodendroglial and glioma stem cell markers do not have an independent prognostic effect in IDH-wildtype glioblastoma.

## Introduction

Glioblastoma (GBM) is the most common malignant brain tumor in adults [1]. Despite increased research efforts during the last decade, the median overall survival remains poor despite a multimodal adjuvant treatment approach after maximal surgical excision [2]. During the last years an abundancy of information about genetic aberrations and subclassifications to identify prognostic factors and therapy response has been published. It is now well established that among high grade gliomas patients with oligodendrogliomas have a much better overall survival when treated according to the correct adjuvant regimen [3,4].

Only little information on the prognostic value of oligodendroglial markers in glioblastoma exists. However, glioma stem cells, which are a driving force for invasion and tumor recurrence in glioblastoma [5], have recently been linked to an oligodendroglial marker. The oligodendrocyte transcription factor 2 (Olig2) was identified as a marker for glioma stem cells [6,7]. It is also highly expressed in other central nervous system tumors such as oligodendrogliomas, pilocytic astrocytomas and diffuse intrinsic pontine gliomas (DIPG) [8–10].

Another biomarker which is regarded as glioma stem cell marker is Nestin, a type VI intermediate filament. It is mainly expressed in precursor neurons and was identified as a marker for glioma stem cells [8]. A few studies have assessed the prognostic value of this marker and showed non-conclusive results [9–12]. Overall, an increased expression seems to have a negative prognostic effect in glioblastoma. However, the differing designs of the studies did not always include all currently established prognostic markers in a multivariate analysis.

Additionally, aquaporin-4 (AQP4), a highly regulated water channel protein in the central nervous system, has been linked to high-grade gliomas as well [13]. More specifically, AQP4 is frequently expressed in GBM and it is widely accepted that an increased expression is associated with glioma invasion and migration [13,14].

In 2012 Marucci et al. described how the neurite outgrowth inhibitor A (Nogo-A) was successfully utilized for the differentiation of oligodendrogliomas from other gliomas [15]. An increased expression was also observed in a subgroup of 24% of GBM [16]. However, no further analysis of the clinical or prognostic impact in GBM has been done according to the current literature.

The prognostic significance of novel markers found in retrospective analyses needs to be validated on the basis of established clinical and molecular prognostic factors in order to allow valid prognostic conclusions. In the present study, we conducted a single center retrospective analysis evaluating the prognostic role of Olig2 as well as other glioma stem cell and oligodendroglial markers in a glioblastoma cohort using a multivariate analysis including established clinical and molecular prognostic markers.

## Materials and methods

### Patient cohort

Between March 1997 and May 2011 in total 456 patients were diagnosed with glioblastoma in the University Medical Center Göttingen. Clinical records were reviewed for information on histopathological results, age, preoperative Karnofsky Performance Status (KPS) and adjuvant treatment. For the extent of resection a categorization into gross total resection (GTR), subtotal resection (STR) and biopsy was done after reviewing the operative reports. Information about patient survival was either acquired by reviewing clinical records or by contacting the treating primary care physician. The study was approved by the local ethics committee. Only cases with full information on age, KPS, overall survival (OS) and adjuvant treatment were considered for this study. Patients, who received similar adjuvant treatment consisting of radiation therapy and chemotherapy with alkylating agents were included. Cases were excluded if the amount of tumor tissue was insufficient for processing into tissue microarrays (TMA). This left 117 eligible patients with GBM that underwent sequencing for *IDH1* and *IDH2* mutations. Subsequently, 4 cases with *IDH*1-R132H mutations were excluded and 113 patients, who were diagnosed with IDH-wildtype glioblastoma WHO grade IV, were left for final analyses. A summary of clinical information of the patient cohort can be found in Table 1.

**Table 1:** Patient cohort characteristics.

	n (%) 113 (100)
**Gender**	
Male	67 (59)
Female	46 (41)
**Age at diagnosis**	
Mean (years)	63
Median (years)	64
Range (years)	24–80
**KPS (preoperative)**	
100%	4 (4)
90%	16 (14)
80%	22 (19)
70%	39 (35)
< 70%	32 (28)
**Overall Survival**	
Median (months)	12.5
Range (months)	2.1–37.3
**MGMT**	
*MGMT* gene promoter methylated	37 (33)
*MGMT* gene promoter unmethylated	71 (63)
*MGMT* gene promoter data missing	5 (4)
**EOR**	
GTR	88 (78)
STR	24 (21)
Biopsy	1 (1)
**Adjuvant CT**	
TMZ	89 (79)
ACNU	16 (14)
BCNU	8 (7)
**Adjuvant RT**	113 (100)

ACNU: nimustine, BCNU: carmustine, CT: Chemotherapy, EOR: extent of resection, GTR: gross total resection, KPS: Karnofsky performance scale, MGMT: O6-methylguanine-DNA methyltransferase, RT: radiotherapy, STR: subtotal resection, TMZ: temozolomide

### Specimen selection and tissue microarray (TMA) preparation

Paraffin-embedded tumor samples from tumor resections and diagnostic biopsies were acquired from the tumor tissue bank of the Institute of Neuropathology of the University Medical Center Göttingen. The corresponding hematoxylin and eosin (HE) stained sections from the donor blocks were microscopically re-evaluated by two independent certified neuropathologists (CS, CH) according to the revised fourth edition of the WHO classification of brain tumors [17]. Areas Suitable for 2 mm sample cylinder extraction were chosen based on the presence of 1) histological diagnostic criteria of glioblastoma and 2) sufficient viable tumor tissue. Cases with inadequate tumor samples were excluded, which was the case for most patients who received a biopsy.

Samples of two selected regions of the donor block were manually extracted using a 2 mm diameter biopsy punch (PFM medical, Germany) and transferred to a 6x10, 2mm Quick-Ray recipient TMA block according to the manufacturer´s instructions (Sakura Finetek, USA). A total of 25 cases were included on each block. Additionally, tissue cylinders containing normal cerebellum, normal hippocampus, oligodendroglioma, pilocytic astrocytoma and a cerebral melanoma metastasis were incorporated as controls. An HE stained tissue section was prepared from each TMA-block and evaluated to confirm the correct transfer of the selected tumor areas.

### Immunohistochemistry

Sections with a thickness of 5μm were prepared from the glioblastoma TMAs using a microtome (Leica SM 2000R, Leica, Germany) and stained using standard histochemical (e.g. HE) and immunohistochemical (IHC) techniques. Olig2 and NogoA were used as oligodendroglial markers and Olig2 and Nestin for glioma stem cell staining. Additionally, AQP4 was applied to provide information on glioma invasion and migration. Shortly, the sections were deparaffinized, pretreated and blocked using 10% fetal calf serum diluted in phosphate buffered saline (PBS) or normal goat serum for Olig2-IHC. For nuclear stainings, a short permeabilization with triton (NogoA) or 0.2% casein (Olig2) was additionally performed. Then tissue sections were incubated overnight at 4°C with primary antibodies against NogoA (1:500, mouse monoclonal, Santa Cruz, USA), Olig2 (1:300, rabbit polyclonal, IBL-America, USA), Aquaporin-4 (1:200, rabbit polyclonal, Sigma Aldrich, Germany) or Nestin (1:100, mouse monoclonal, Millipore, Germany). Suitable biotinylated secondary antibodies (GE Healthcare, Germany and Dianova GmbH, Germany) followed by 3,3′-diaminobenzidine hydrochloride (DAB) developed avidin-peroxidase were used for antibody detection. Nuclei were visualized by hematoxylin counterstaining. All 113 tumor samples were analyzed for NogoA and Olig2 while a few cases were lost due to IHC methodological issues for AQP4 (4) and Nestin (7). Individual photographs of TMA samples were taken with a XM10 camera (Olympus, Germany) mounted on a BX51 light microscope (Olympus, Germany) using 4x, 10x, 20x or 40x objective lenses. For image acquisition the cellSense Dimension 1.7.1. software was used (Olympus, Germany).

### Sequencing of IDH1, IDH2 and MGMT promoter methylation status

DNA extraction from FFPE tissue was done with the DNeasy Blood & Tissue Kit (Qiagen, Hilden, Germany) and DNA concentration assessed with the FLUOstar Omega plate reader photometer (BMG LABTECH GmbH, Ortenberg, Germany). The mutation status of *IDH1* and *IDH2* was evaluated by Sanger sequencing. For this purpose 50 ng of tumor DNA was mixed with 10 μ of HotStar Taq 2X Mastermix (Qiagen), 1 μl primers (forward and reverse) and filled to a final volume of 20 μl with high purity water (PCR primers—IDH1 forward: CGGTCTTCAGAGAAGCCATT, IDH1 reverse: GCAAAATCACATTATTGCCAAC, IDH2 forward: AGCCCATCATCTGCAAAAAC, IDH2 reverse: CTAGGCGAGGAGCTCCAGT). Thirty cycles of denaturation at 95°C for 30 seconds each were followed by annealing at 60°C for 30 seconds and elongation at 72°C for 60 seconds were completed with final elongation for 10 minutes at 72°C. Agarose gel electrophoresis was applied for verification. The PCR products (5 μl) were incubated with Illustra ExoProStar OneStep enzyme mix (5 μl) (GE Healthcare, Buckinghamshire, UK) at 37°C for 20 minutes followed by 15 minutes of inactivation at 80°C. The *IDH1/2* primers were added, and the samples sent to an external sequencing service provider (GATC, Konstanz, Germany).

Methylation of the *MGMT* promoter was determined by pyrosequencing as described by Banan et al. [18]. Tumor DNA underwent bisulfite conversion with the EZ DNA Methylation-Gold-Kit (Zymo Research, Irvine, CA, USA) and was then used as PCR template. Bisulfate converted DNA (100 ng), Platinum Taq Polymerase (0.1 μl) (Thermo Fisher Scientific, Waltham, MA, USA), PCR primers (1 μl each) 10mM dNTP (1 μl) (Thermo Fisher Scientific), PCR buffer (2.5 μl) and 25 mM MgCl2 (2.5 μl) were mixed for PCR amplification and filled to a final volume of 25 μl with high purity water (PCR primers: MGMT_1i5’: GTTTYGGATATGTTGGGATAG, MGMT_3i3’: AACCACTCRAAACTACCACC, MGMT_3i3’bio: Biotin-AACCACTCRAAACTACCACC and MGMT_1i5’bio: Biotin-GTTTYGGATATGTTGGGATAG). An equimolar mix of oligonucleotides with C/T (Y) or G/A (R) nucleotides at the position of CpG sites was used since *MGMT* PCR primers span CpG sites which may have been modified by bisulfite conversion. After initial activation at 95°C for 5 minutes, 45 cycles consisting of denaturation at 95°C for 30 seconds, annealing at 60°C for 45 seconds and elongation at 72°C for 30 seconds were completed, finalized with 5 minutes of elongation at 72°C. Agarose gel electrophoresis was applied for product verification. The PyroMark Q96 MD system with PyroMark Gold Q96 reagents (Qiagen) were used for pyrosequencing together with the primers MGMT-pyro1: TGGTGAGTGTTTGGGT and MGMT-pyro1R: CCAAACACTCACCAAAT. 5–10 μl of the PCR product and streptavidin-coated sepharose beads were mixed for strand separation and subsequent washing (vacuum prep tool, Qiagen). Annealing was performed by adding 5 μl of the sequencing primer (0.5 μM) to the bead-bound ssDNA at 80°C for 2 minutes and final cooling at room temperature. PyroMark MD software (Qiagen) was used to analyze the sequencing results. A mean methylation value above 10% was considered as “hypermethylated” [19].

### Microscopic evaluation and statistical analysis

The relative abundance of IHC markers was determined in a semi quantitative manner by two observers blinded to clinical data. Inconclusive cases were reviewed, and a consensus was achieved. For IHC of the nuclear protein Olig2, the percentage of positive cells was visually estimated for each sample and its average was coded as an ordinal variable with 8 levels (Table 2). Analogously, for IHC of cytoplasmic marker proteins (AQP4, Nestin, NogoA) the area of positive staining was estimated for individual samples according to the scoring system presented in Table 2. The appropriate scoring system was chosen according to the staining characteristics of the IHC marker. An intensity score was not applied.

**Table 2:** Scoring system for immunohistochemical evaluation.

	Score							
	0	1	2	3	4	5	6	7
AQP4	Negative	<1%	1–5%	>5–50%	>50%			
Nestin	Negative	<1%	1–5%	>5–50%	>50%			
NogoA	Negative	<1%	1–5%	>5–50%	>50%			
Olig2	0–5%	>5–10%	>10–20%	>20–30%	>30–40%	>40–50%	>50%-60%	>60%

Percentage of immunopositive cells estimated by microscopic evaluation.

Dichotomization at different cut offs for each marker was done for univariate analysis to identify a possible impact on OS or correlation with other clinical aspects (gender, age, KPS). Established prognostic factors (age, KPS, and *MGMT* promoter methylation status) were assessed as possible covariates. Statistical significance was defined as α<0.05. Pearson’s Chi-squared test for the analysis of categorical variables was applied. Univariate survival analysis was done with ANOVA analysis of variance and Kaplan-Meier curves with Log-rank testing. Multivariate Cox regression was applied including established prognostic factors (age, KPS and *MGMT*-promoter methylation status) and immunohistochemical factors that showed a significant univariate impact on OS. A Classification and Regression Tree analysis (CART-analysis) was done for age and the corresponding cut off was used for further analyses. All statistical analyses were performed using JMP® (Cary, NC: SAS Institute Inc.; 1989) Statistical Discovery Software, version 13.1.1.

### Ethics committee

The study was approved by the Ethics Committee of the University Medical Center Göttingen (No 24/10/05, Amendment 21/3/11).

## Results

### Patient cohort characteristics

After exclusion of cases with tumor tissue unsuitable for TMA processing, incomplete datasets or *IDH1/2* mutation, 113 patients with IDH-wildtype GBM were analyzed. The median age at diagnosis was 64 years, ranging from 24 to 80 years. The male to female ratio was 3:2 and the median overall survival 12.5 months. Seventy-two percent had a preoperative KPS of 70%. In one third a *MGMT* gene promoter methylation was detected, while 63% were unmethylated and 4% could not undergo analysis due to lack of sufficient tumor tissue. All patients received adjuvant radiotherapy and chemotherapy with an alkylating agent (see Table 1).

### Gender, KPS, age and MGMT-promoter methylation status

There was no impact of the patients’ gender on overall survival or other clinical parameters (age, clinical status, expression of immunohistochemical markers). The established prognostic factors KPS and age at diagnosis as well as the *MGMT*-promoter methylation status showed a significant prognostic impact in our patient cohort (see multivariate analysis results in Table 3). The CART analysis revealed a cut off at 65 years of age to be most fitting for splitting the patient cohort in regard of difference in OS (65.22 years). The ANOVA and Log-rank test showed a significantly longer OS for younger patients (17.2 compared to 11.6 months/p = 0.0038 and 15.3 months compared to 10.1 months / p = 0.0091, respectively) and also in the multivariate Cox regression (p = 0.0113). Other cut offs at 50, 60 and 70 years were assessed as well, but did not show a significant impact on overall survival. The widely used KPS cut off at 70% was applied and slightly missed the level of significance in the ANOVA (15.6 compared to 11.5 months, p = 0.0564), while a significant survival advantage for patients with a better clinical status was seen in the univariate (Log-rank: 13.4 months compared to 11.1 months, p = 0.031) and multivariate analysis (p = 0.0320). One third of the patient cohort had methylated *MGMT*-promoters and a significantly better mean overall survival (ANOVA: 16.3 compared to 12.5 months, p = 0.0191) Uni- (14.8 months compared to 11.7 months, p = 0.0193) and multivariate survival analysis (p = 0.0152) confirmed this. The summarized results of the survival analyses are displayed in Table 3.

**Table 3:** Survival analysis.

		Univariate (ANOVA)	Univariate (Log-rank)	Multivariate
	n (%)	Mean OS (95%CI)	Median OS (95%CI)	Cox Regression
**Gender**	113 (100)			
Male	67 (59)	14.8 (12.4–17.4)	13.4 (11.8–16.1)	
Female	46 (41)	13.8 (10.8–16.8)	11.4 (8.0–13.1)	
		p = 0.5835	p = 0.3252	
**Age**	113 (100)			
> 65	56 (50)	11.6 (9.0–14.3)	10.1 (7.2–11.8)	
< 65	57 (50)	17.2 (14.5–19.8)	15.3 (12.5–16.7)	
		p = 0.0038*	p = 0.0091*	p = 0.0113*
**KPS**	113 (100)			
>/ = 70	81 (72)	15.6 (13.3–17.8)	13.4 (11.7–15.6)	
< 70	32 (28)	11.5 (7.9–15.0)	11.1 (7.3–12.9)	
		p = 0.0564(*)	p = 0.0301*	p = 0.0320*
**MGMT gene promoter**	108 (100)			
methylated	37 (34)	16.3 (13.7–18.9)	14.8 (11.4–20.5)	
unmethylated	71 (66)	12.5 (10.6–14.3)	11.7 (10.4–13.3)	
		p = 0.0191*	p = 0.0193*	p = 0.0152*
**NogoA**	113 (100)			
Positive	68 (60)	14.3 (11.8–16.8)	12.6 (11.1–13.8)	
Negative/single cells	45 (40)	14.6 (11.8–16.8)	12.4 (10.4–15.9)	
		p = 0.8539	p = 0.6611	
**Olig2 (cut off 10%)**	113 (100)			
High	89 (79)	13.2 (11.1–15.3)	11.8 (10.9–13.5)	
Low	24 (21)	18.9 (14.8–22.9)	14.0 (10.4–22.5)	
		p = 0.0163*	p = 0.0642 (*)	p = 0.1194
**AQP4 (cut off 5%)**	109 (100)			
High	86 (79)	14.0 (12.2–15.7)	12.6 (11.4–14.2)	
Low	23 (21)	14.4 (10.0–16.8)	10.4 (7.2–17.7)	
		p = 0.7646	p = 0.7554	
**Nestin (cut off 5%)**	109 (100)			
High	65 (60)	14.4 (12.4–16.4)	12.8 (11.1–14.8)	
Low	44 (40)	13.0 (10.5–15.4)	11.8 (9.4–15.6)	
		p = 0.3538	p = 0.2488	

KPS: Karnofsky performance scale, MGMT: O6-methylguanine-DNA methyltransferase OS: overall survival.

### Immunohistochemical findings

**Fig 1:**
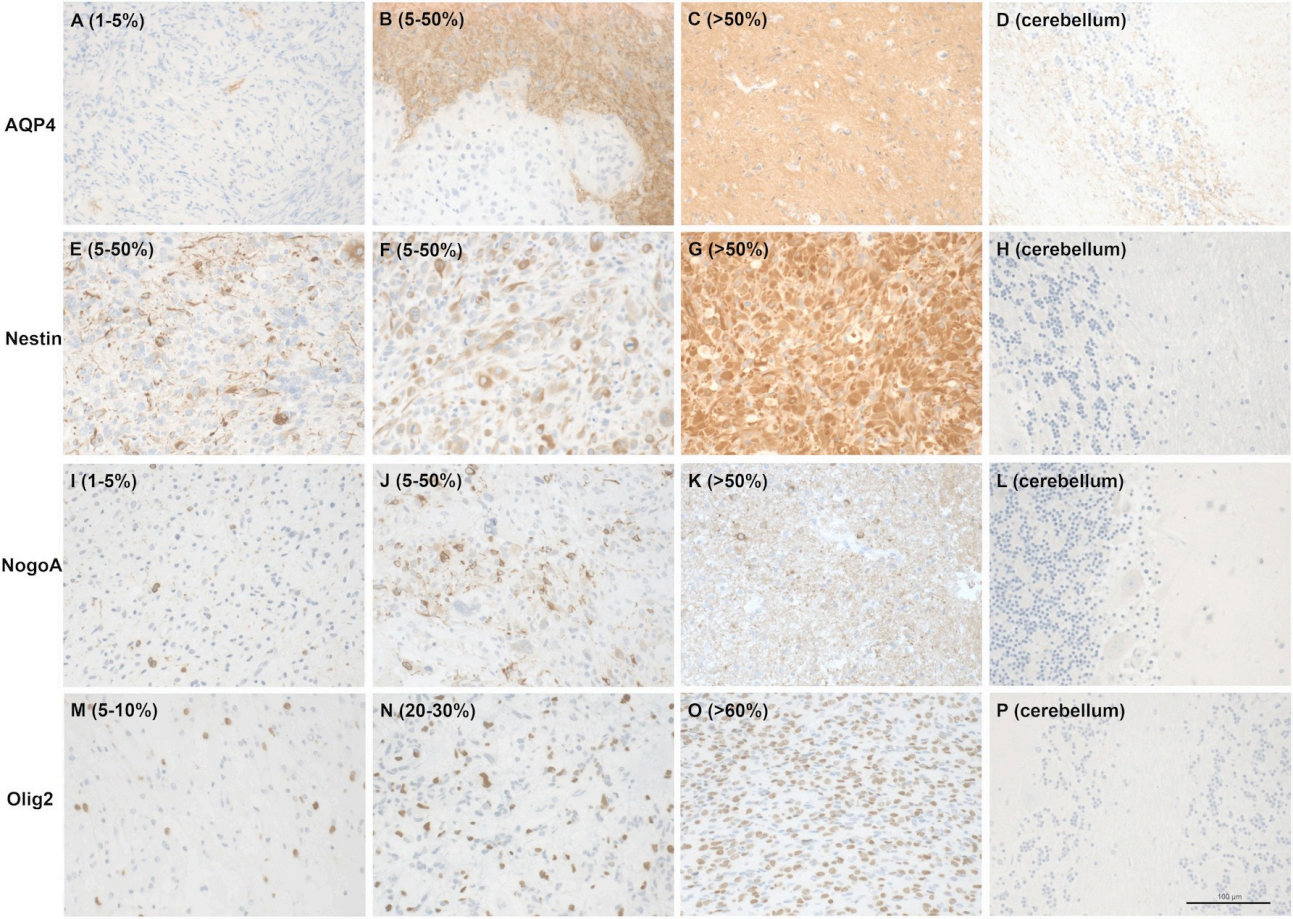
Images of patient samples as examples of the semiquantitative immunohistochemical scoring system for AQP4 (A: 1–5%, B: 5–50%, C: >50%), Nestin (E: 5–50%, F: 5–50%, G: >50%), NogoA (I: 1–5%, J: 5–50%, K: >50%) and Olig2 (M: 5–10%, N: 20–30%, O: >60%). Non-neoplastic cerebellar tissue (D, H, L and P) was used as a negative control for each staining. Magnification: 200x.

**Fig 2:**
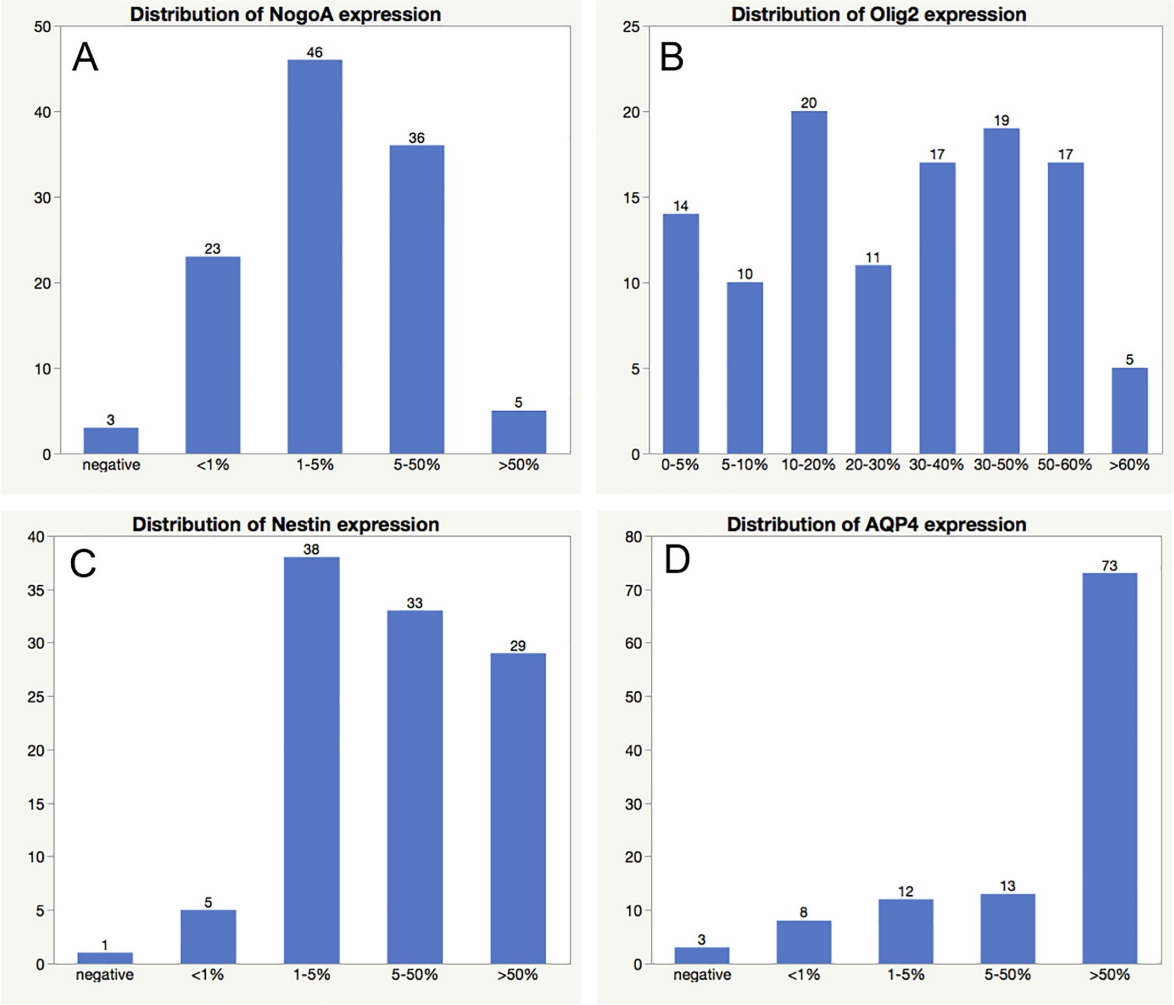
Distribution of the different immunohistochemical scores of each marker (A: NogoA, B: Olig2, C: Nestin, D: AQP4).

**Fig 3:**
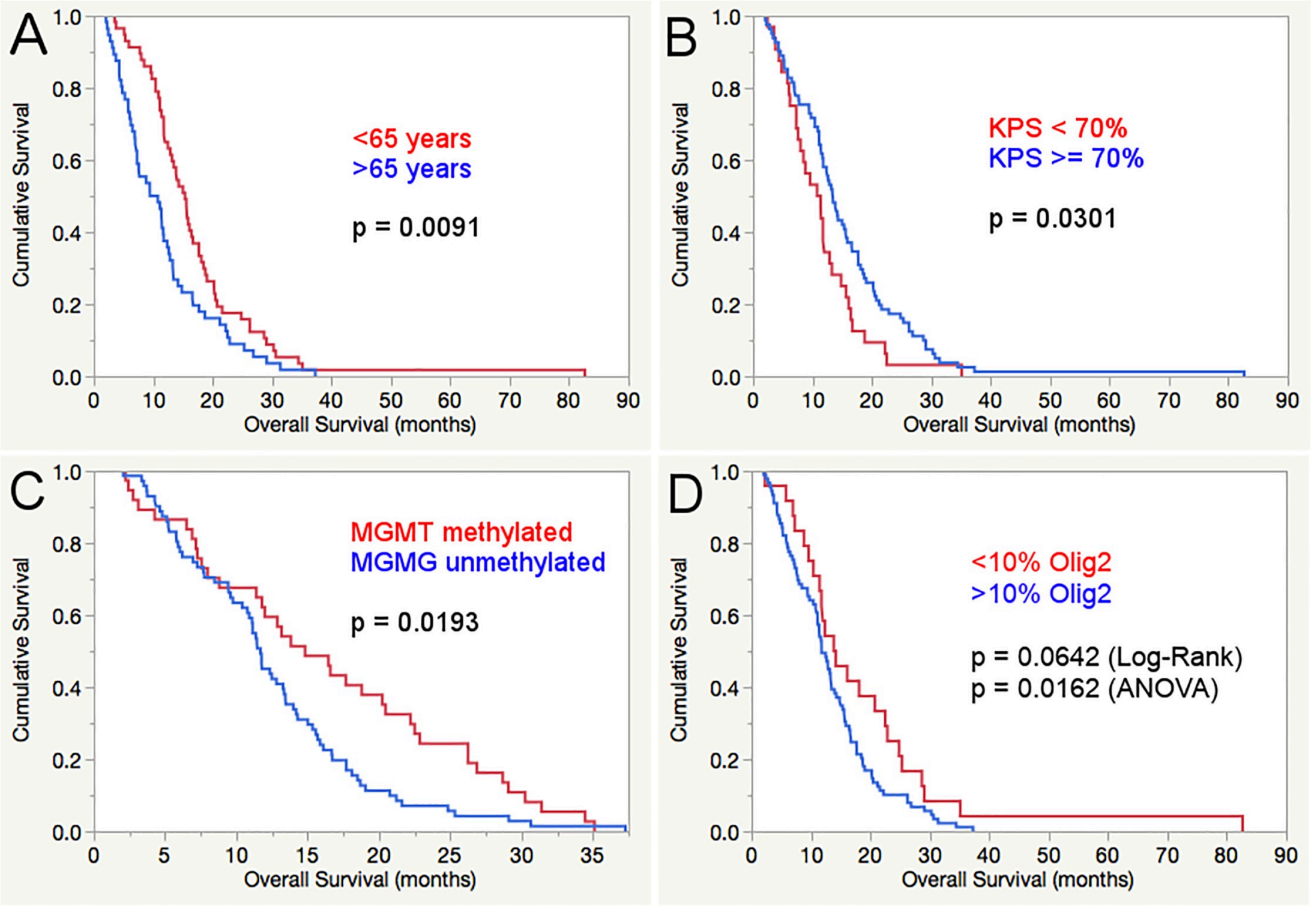
Kaplan-Meier curve and Log-rank test displaying the significant prognostic impact of age below 65 years (A), better clinical status (KPS >/ = 70) (B) and methylated *MGMT*-promoter status (C) as well as a trend towards survival benefit of Olig2 expression below 10% in the Log-rank test and a significant difference in the ANOVA (D).

The staining and distribution of all assessed immunohistochemical markers is displayed in Figs 1 and 2. Each marker was evaluated at different cutoffs regarding impact on OS and clinical characteristics (gender, age, KPS). No significant differences in OS, age, KPS and gender were observed for the assessed cut offs for NogoA, AQP4 and Nestin (see Table 3 and Fig 3).

**Fig 4:**
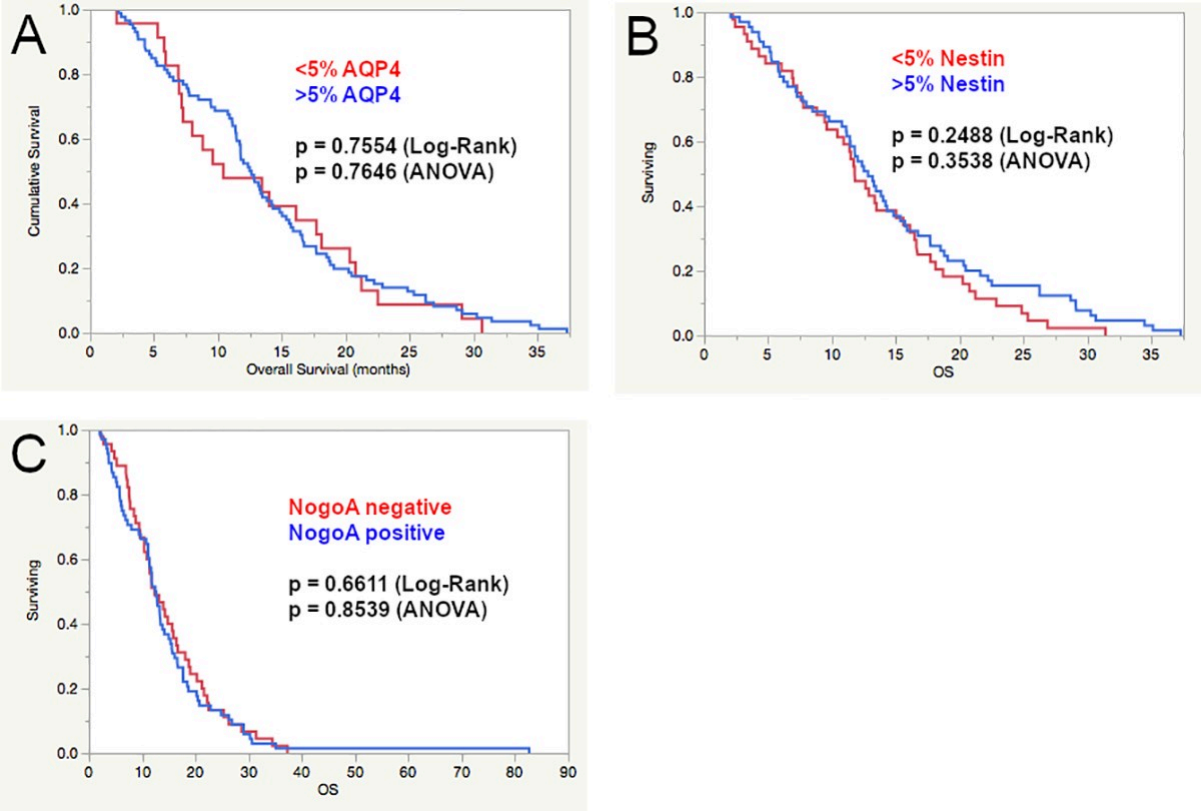
Kaplan-Meier curves and Log-rank test of AQP4 (A), Nestin (B) and NogoA (C). No significant differences were observed. For comparison the univariate p-value of the ANOVA is provided.

Higher expression of Olig2 showed a significant negative effect on OS at different cut offs (ANOVA, see Table 3). At the 5% cut off, patients with lower expression had a mean OS of 20.6 months (15.2–25.9) compared to 13.5 months for patients with higher expression (11.5–15.6) (p = 0.0161). Similar results were seen at the 10% cut off (18.9 months (14.8–22.9) compared to 13.2 (11.1–15.3), p = 0.0163). At the cut offs at 20 and 30% a non-significant trend towards longer OS with lower Olig2 expression was observed (p = 0.0733 and 0.0913, respectively). In the univariate survival analysis (Log-rank test, see Fig 4) there was no statistical significance at all cut offs for Olig2, while a non-significant trend was observed at the 10% cut off (14.0 months compared to 11.8 months, p = 0.0642). In the multivariate Cox regression analysis, we assessed each Olig2 cut off together with the established prognostic factors age (cut off 65 years according to the CART analysis), KPS (>/ = 70%, according to the widely used cut off in the literature) and *MGMT*-promoter methylation status. No significant independent impact on OS of any of the Olig2 cut offs was seen. There was no difference in age, KPS, gender or *MGMT*-promoter methylation status at each Olig2 cut off.

## Discussion

We evaluated a representative cohort of patients diagnosed with IDH-wildtype glioblastoma WHO grade IV, displaying similar clinical characteristics (distribution of gender, age, clinical status) as described in the literature [20]. All patients received adjuvant radiotherapy and chemotherapy with an alkylating agent. Consistent with previous reports, patient age and clinical status as well as *MGMT*-promoter methylation status were all significant independent prognostic factors in the multivariate analysis [21–23].

Unfortunately, all glioblastoma patients eventually died due to their disease during the course of the study. Therefore, our data reached maximal maturity, meaning that survival data of all patients is complete without censored survival data. This explains that all Kaplan-Meier curves eventually show crossing of both groups in the later course, as to be expected with maximally mature survival data in a fatal disease as GBM.

### Oligodendroglial markers

The information on the clinical impact of Nogo-A expression in GBM is scarce. Kuhlmann et al. were able to show that a subgroup of 24% of GBM have an increased expression of Nogo-A [16]. In 2012 Nogo-A was applied to differentiate oligodendrogliomas from other glioma types, raising the hope that this marker might be able to discriminate a new subgroup of GBM [15]. Our findings revealed a heterogeneous expression of Nogo-A in GBM but without correlation with age, clinical status, *MGMT*-promoter methylation status or overall survival.

Nestin on the other hand has been assessed more extensively in gliomas. It was identified as a glioma-stem cell marker [8] and several studies focused on the prognostic effect in different glioma subtypes [9–12]. In 2014 Dahlrot et al. presented a retrospective study assessing the prognostic impact of Nestin in different glioma subtypes in a multivariate analysis including established prognostic markers. The authors found no impact of Nestin expression on overall or progression-free survival in GBM and proclaimed that the differing results in prior studies could be explained by suboptimal statistical models without the inclusion of other prognostic markers [9]. Our study takes this idea one step further by the inclusion of the *MGMT*-promoter methylation status, exclusion of IDH mutated GBMs and uniform adjuvant treatment (patient receiving radiation and chemotherapy only), which was not considered in the analysis by Dahlrot et al.. We can confirm that Nestin expression has no independent prognostic impact in IDH-wildtype GBM. However, it must be noted that the tumor volume and the extent of surgical resection, another established strong prognostic factor, were not included in our study, due to the lack of complete volumetric data.

In 2007 Warth et al. analyzed the prognostic role of AQP4 expression in different gliomas and found an exceptionally high expression in pilocytic astrocytomas WHO grade I and GBMs but without any prognostic value [24]. Since then, extensive research has been done regarding the role of AQP4 in GBM and its important function in tumor cell migration and proliferation is now well established [13,14]. An increased effort is put into the development of possible AQP4 inhibitors, so far without success [13]. Our data contribute to this topic by showing the distribution of AQP4 expression in paraffin-embedded tumor tissue of IDH-wildtype glioblastomas and revealing no differences in age, clinical status, MGMT-promoter methylation and prognosis at different expression cutoffs.

The expression of Olig2 was associated with significantly shorter overall survival in the univariate analysis of our patient cohort at the cut offs 5 and 10% (ANOVA). However, statistical significance was lost in univariate Log-Rank test as well as in the multivariate analysis when including established prognostic markers (age, KPS and *MGMT*-promoter methylation status). There are several reasons that might explain the lack of a significant independent effect in the multivariate analysis. In our study, we excluded cases in which only biopsies were performed or cases in which the amount of available tumor tissue for TMA construction was insufficient. Therefore, cases with an especially grim prognosis, due to a tumor location not amenable to surgical resection or poor clinical status are underrepresented. It is therefore possible that less stringent selection criteria and a larger sample cohort may show different results.

Nevertheless, the significant negative impact of higher Olig2 expression at multiple cut offs in the ANOVA analysis cannot solely be explained by prognostic confounding factors like differences in clinicals status (KPS), age or *MGMT*-promoter methylation status of the patient groups created by the respective cut offs. Olig2 is expressed in 70–80% of diffuse intrinsic pontine gliomas (DIPG), a pediatric aggressive brainstem glioma, and regarded as an important transcription factor in gliomagenesis [25]. It is also a highly-expressed biomarker in diffuse gliomas and pilocytic astrocytomas [26,27]. Prior to that, Olig2 was identified as a significant marker for glioblastoma stem cells (GSC) [7]. GSC are highly therapy-resistant cancer stem cells that play a major role in tumor recurrence and invasion [28]. The role of Olig2 in propagating tumor growth and the antiproliferative effect of Olig2 deletion was recently shown [29]. Thus, a trend towards negative prognostic impact of higher Olig2 expression could be explained by the association with a higher frequency of glioma stem cells and consequently, a worse prognosis.

Another important issue in glioma research is the non-unified tumor sampling. Usually there is insufficient information on the exact localization where different tumor samples originate from (e.g. glioma tissue near the cortex or the subventricular zone (SVZ)). It has been well described how GSC reside in the SVZ and the deep-seated areas of gliomas [30], locations that are more likely to be left unresected due to its proximity to eloquent areas (basal ganglia and pyramidal tract) or the preference to leave the ventricular system intact, in fear of enabling tumor cell spread via the cerebrospinal fluid and hydrocephalus development [31]. However, it has been shown that the relation of the tumor lesion to the SVZ has a prognostic value [32–34].

It will be crucial in the future to provide a more detailed sampling description to allow a differentiated analysis of glioma tissue, especially in the light of the prognostic role of glioma-stem cells in tumor invasion and recurrence. It is of high clinical interest to be able to identify GSC histopathologically but also on preoperative scans or even intraoperatively. To further understand the mechanism of glioma recurrence and invasion it will also be crucial to assess glioma stem cell markers comparatively in primary and recurrent tumors.

### Limitations

Overall the retrospective nature of the study is always prone to a selection bias. Since overall survival was the primary endpoint, we have excluded patients that did not receive radiation therapy and chemotherapy with an alkylating agents. Furthermore, based on the amount of tissue that is necessary to construct tissue microarrays, most tumors that were only biopsied were not suitable for this method. Thus, only one biopsied glioblastoma was included and patients with reduced clinical status or tumors of unfavorable location and size may be underrepresented in this cohort, which can be important when assessing the prognostic impact of biomarkers and may explain the negative results shown in this study. However, tumor size/location as well as clinical status that may have led to the choice of biopsy over resection are very unlikely related to the tumor biology and thus the underrepresentation of such cases should have no impact on the study outcome. Additionally, the data of extent of resection is based on the surgeon’s postoperative note and is not based on an exact volumetric measurement.

## Conclusions

Different expression levels of Olig2, Nogo-A, Nestin and AQP4 have no independent prognostic impact in IDH-wildtype glioblastoma and show no distribution differences regarding age, clinical status and *MGMT*-promoter methylation status.

## Supporting information

S1 Table (XLSX)
